# When Educational Images Don’t Reflect the Population: Phlegmasia Cerulea Dolens, a Case Report

**DOI:** 10.5811/cpcem.1905

**Published:** 2024-04-17

**Authors:** Kasha Bornstein, Elizabeth LaRosa, Kelsey Byrd, Dan Laney, Hector Ferral, Heather Murphy-Lavoie

**Affiliations:** * Louisiana State University Health Sciences Center, Department of Internal and Emergency Medicine, New Orleans, Louisiana; † Louisiana State University Health Sciences Center, Department of Emergency Medicine, New Orleans, Louisiana; ‡ Louisiana State University Health Sciences Center, Department of Interventional Radiology, New Orleans, Louisiana

**Keywords:** *case report*, *health disparities*, *deep venous thrombosis*

## Abstract

**Introduction:**

Phlegmasia cerulea dolens (PCD) is an uncommon, potentially life-threatening complication of acute deep venous thromboses that requires a timely diagnosis. The name of the condition, the visual diagnostic criteria, and the preponderance of cases in the literature referencing findings exclusively in patients with lighter skin complexions means that PCD may not be on the differential diagnosis for the patient with more melanated skin who is experiencing this time-sensitive vascular emergency.

**Case Report:**

We describe one case of PCD in a patient with darker skin complexion and the importance of identifying clinical findings, regardless of skin color, given the paucity of reference images for PCD in darker complected patients. Our literature review yielded 60 case reports for PCD. Only two papers included images referencing patients of color.

**Conclusion:**

Accurate diagnosis requires recognition of diagnostic findings, which may vary significantly between phenotypically distinct populations. Many pathognomonic physical exam findings rely on descriptors based on presentation in phenotypically white patients.

CPC-EM Capsule What do we already know about this clinical entity?

*Phlegmasia cerulea dolens (PCD) is a potentially life-threatening sequela of deep vein thrombosis characterized by extremity swelling, pain, and skin color change.*

What makes this presentation of the disease reportable?

*We describe a case of PCD in a patient with darker complexion, emphasizing distinctions in exam findings.*

What is the major learning point?

*Physicians are not primed to visually diagnose conditions in patients with darker-complected skin, as the literature almost uniformly references findings in White patients.*

How might this improve emergency medicine practice?

*Phenotypic differences confer significant variation upon exam findings. Diagnosis requires recognition of pathology between distinct populations.*

## INTRODUCTION

Phlegmasia cerulea dolens (PCD) is a rare and potentially life-threatening complication of acute deep venous thrombosis (DVT), especially if delayed in diagnosis. It is characterized by marked extremity swelling, pain, and skin color change. Additional findings include non-palpable distal pulses and paresthesias.^1^ Phlegmasia cerulea dolens results from critical thrombosis of both the deep and superficial venous systems of the limb, leading to arterial ischemia, gangrene, and limb loss. The mortality rate for PCD ranges between 20–40%; lethal complications include pulmonary emboli (PE), rhabdomyolysis, fluid sequestration, hyperkalemia, and shock.^2^ Even with aggressive intervention, amputation rates are between 12–50%.^3^ The existing literature has a paucity of reference images for PCD in patients with darker skin tones. We describe one case of PCD in a patient with a darker skin complexion, highlighting distinctions in exam findings in patients of color.

## CASE REPORT

A 68-year-old man with a history of pulmonary adenocarcinoma presented to the emergency department (ED) reporting progressive generalized weakness and right lower extremity pain for 10 days. One month prior, he had been hospitalized for shortness of breath and diagnosed with a malignant pleural effusion. This was managed with a tunneled pleural catheter for continuous drainage. During that hospitalization, he was found to have bilateral DVTs of the posterior tibial veins and segmental emboli to the left pulmonary artery. He was started on anticoagulation at that time but subsequently developed hemorrhagic pleural drainage. Anticoagulation was held, and interventional radiology (IR) placed an inferior vena cava (IVC) filter. He was discharged home to continue oncologic treatment.

**Image 1.**
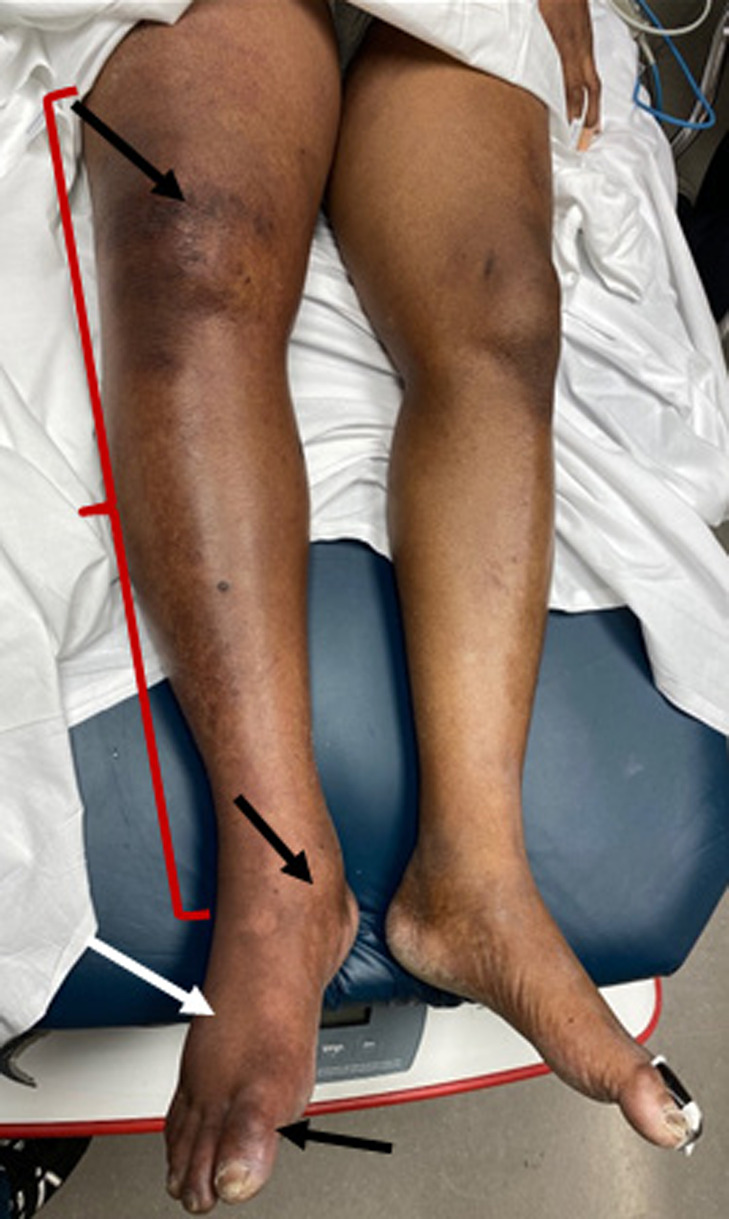
Patient’s right leg with evidence of enlargement (red bracket), darkening of skin around joints (black arrows), subtle reddening and purpling of tissues (white arrow).

Upon his second ED presentation, his exam was significant for sinus tachycardia, normoxemic tachypnea, and hypotension. The skin around the right pleural catheter was clean, dry, and non-erythematous. Pleural catheter drainage was scant and clear. Pulmonary exam was otherwise unremarkable without adventitious lung sounds. His right lower extremity had circumferential, non-pitting edema from the foot to the groin, with areas of erythema, mottling, and purpling about the digits, shin, and knee. His right foot was darkened and cool to touch (Image 1).

**Image 2.**
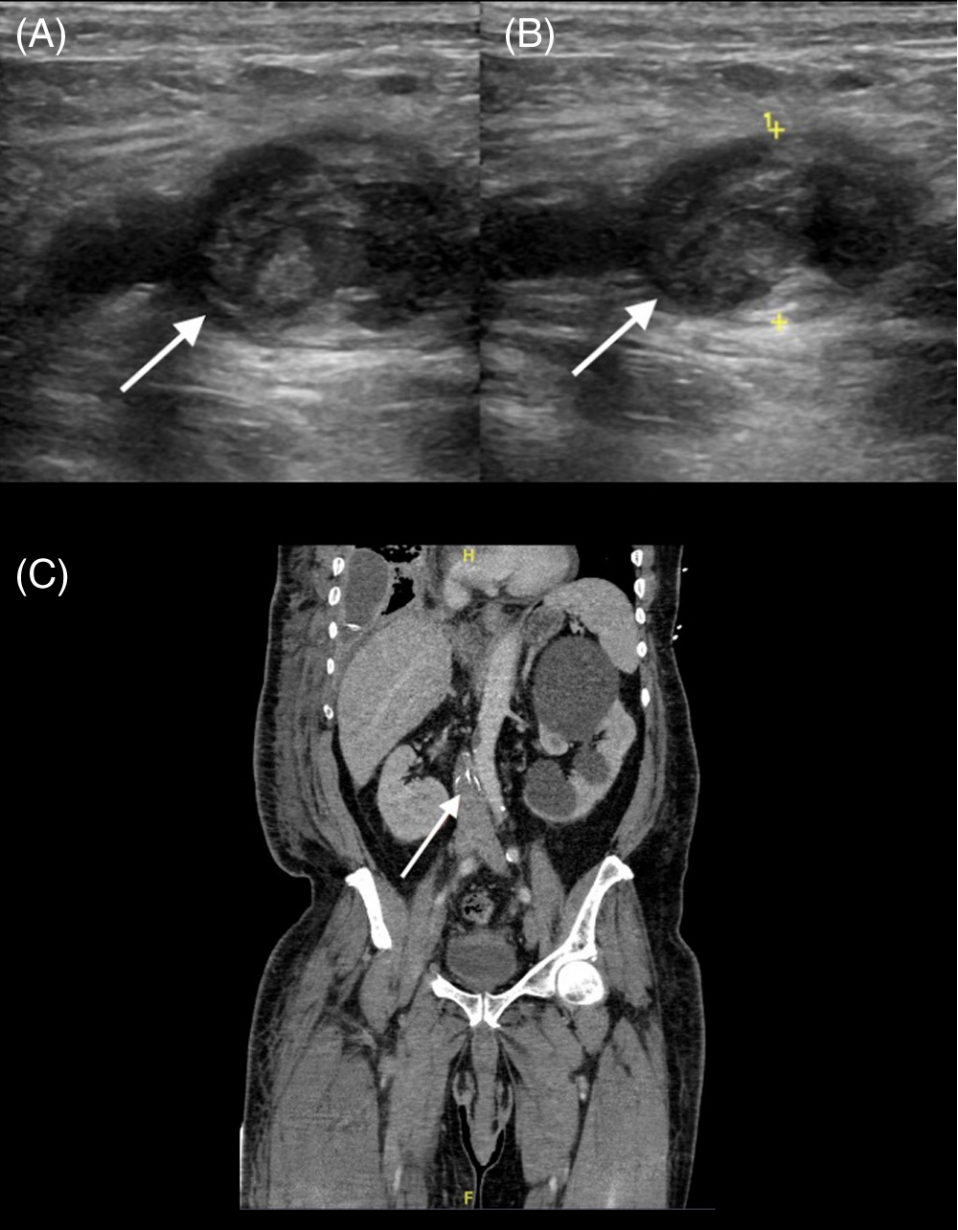
Top: Ultrasound of the right common femoral vein demonstrating clot (A) with incompressibility of the vein (B). Bottom: Computed tomography of the abdomen and pelvis demonstrating thrombosis of the inferior vena cava to the level of implanted inferior vena cava filter (C).

Popliteal, posterior tibial, and dorsalis pedis pulses were palpable. He endorsed pain on passive motion, paresthesias, and decreased sensation distal to the knee. He was able to flex and extend at the hip joint but had complete paralysis below the knee. His left lower extremity was warm and well-perfused, with no edema or neurologic abnormalities. The remainder of his physical exam was unrevealing. Laboratory analyses were notable for normocytic anemia with hemoglobin of 7.7 grams per deciliter (g/dL) (reference range 13.2–16.6 g/dL), consistent with his prior admission, leukocytosis of 12.8 × 10^3^ per microliter (μL) (4.5–11 × 10^3^/μL), potassium of 5.5 millimoles per liter (mmol/L) (3.5–5 mmol/L), creatinine of 1.76 milligrams (mg)/dL (0.74–1.35 mg/dL), up from 1.22 mg/dL on prior discharge. Lactate was elevated to 2.7 mmol/L (reference range <2). COVID-19 polymerase chain reaction was negative. Lower extremity duplex ultrasonography was performed, demonstrating complete thrombosis of the right common femoral, greater saphenous, popliteal, and calf veins (Image 2A, 2B).

Additional thrombi were identified in the left femoral, popliteal, and upper calf veins. This clot burden was greater than had been seen during the prior admission; ultrasound at that time revealed thrombi in the right peroneal and the left popliteal veins. Computed tomography (CT) of the chest revealed no evidence of PE. Computed tomography of the abdomen and pelvis demonstrated extensive bilateral ilio-femoral DVT extending to the IVC filter (Image 2C).

**Image 3.**
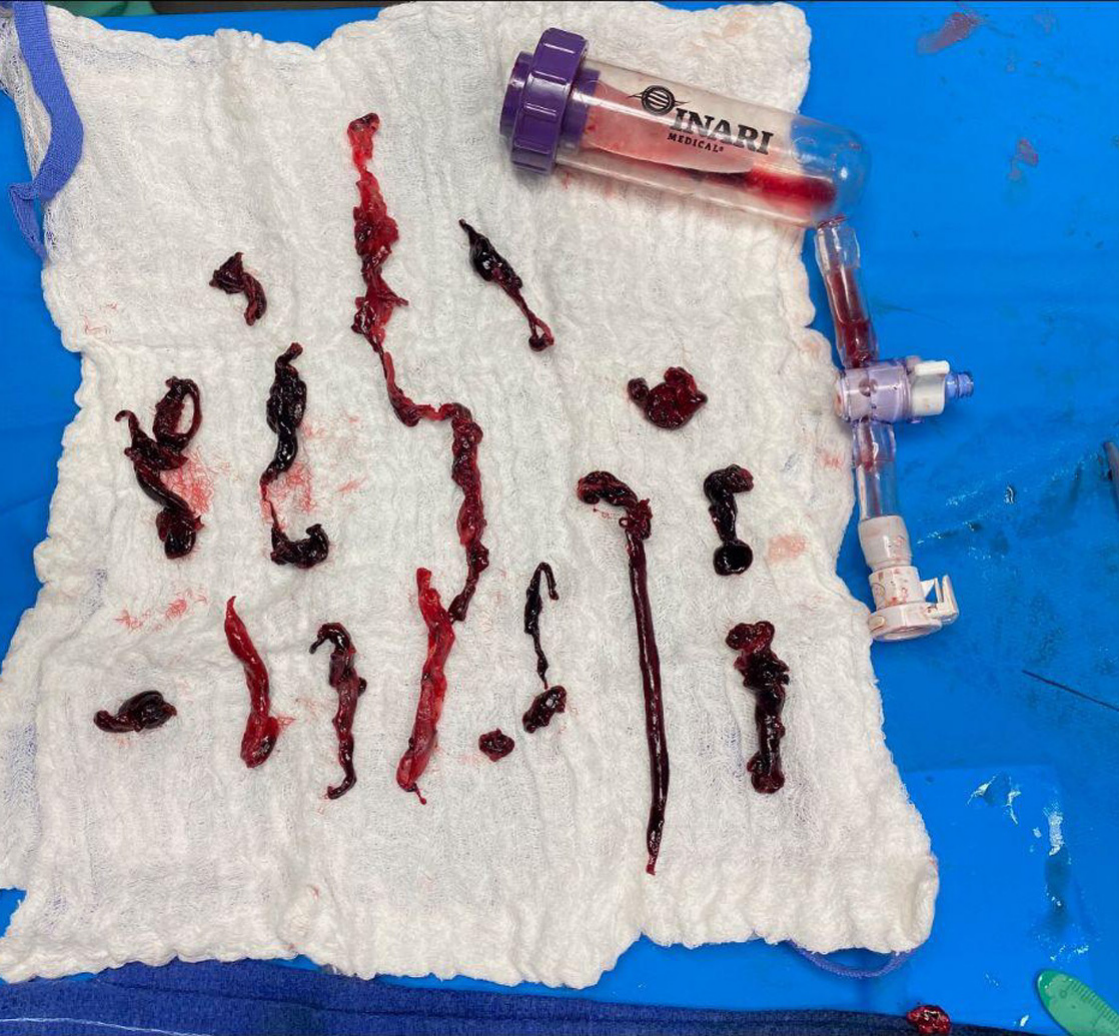
Demonstration of clot burden following thromboaspiration by Interventional radiology service using clot retriever device.

The patient was fluid resuscitated and started on a heparin infusion. Interventional radiology was consulted and performed percutaneous venous thromboaspiration from the popliteal veins to the IVC. Flow was successfully restored throughout the right lower venous vasculature, and 70% of the thrombotic burden was aspirated from the IVC (Image 3). Following intervention, the patient was admitted to the intensive care unit for continued management of hypotension and anticoagulation. Right lower extremity discoloration, edema, and paresthesias resolved within 24 hours following intervention. His hemodynamics improved, and he was transferred to Internal Medicine. He was discharged to a skilled nursing facility on hospital day 15.

## DISCUSSION

Phlegmasia cerulea dolens is an uncommon, life-threatening manifestation of DVT, resulting from critical thrombosis of the deep and superficial venous systems leading to arterial compromise and decreased perfusion. Risk factors for PCD are similar to other DVT and include malignancy, hereditary coagulopathies, surgery, trauma, IVC filter placement, pregnancy, smoking, and use of hormonal contraception.^4^^,^^5^ Unlike simple DVT, PCD also carries risk of compartment syndrome, rhabdomyolysis, electrolyte disturbances, tissue ischemia, gangrene and limb loss, fluid sequestration leading to hemodynamic compromise, and consequent multiorgan failure.^3^

Recognizing this serious condition is essential. Exam findings suggestive of PCD include darkening of the limb(s), extremity edema, decreased skin sensation, paresis, and muscular or superficial tenderness. Crucial physical exam components include inspection and palpation of the entire extremity to evaluate arterial pulses, temperature, and capillary refill. A detailed neurological exam should assess sensory and motor function of the limb. Early duplex ultrasound is critical for assessing non-compressible veins from the distal to proximal vasculature of the affected extremity. As a time-sensitive condition, angiography and other vascular imaging is unnecessary unless suspicion is raised for concomitant PE. Management includes hemodynamic stabilization, treatment of electrolyte derangements, initiation of anticoagulation, and vascular surgery or IR consultation for thrombectomy.^2^

Accurate diagnosis and management of life- or limb-threatening conditions is an essential role of emergency physicians. We believe this case illustrates that this objective was achieved in the care of this patient. However, our aim was not simply to illustrate a case of PCD. We present this case to emphasize the potential consequences of medical education resources that insufficiently depict PCD in non-white patients. Although this patient had many of the risk factors and classic physical exam findings of PCD, (active malignancy, presence of an IVC filter, extremity pain, and paresis) the diagnosis was briefly delayed by difficulty recognizing these features in a patient whose leg physiologically could not be blue. By virtue of the condition’s name—painful blue inflammation—physicians are not primed to visually diagnose this condition in darker-complected skin, as educational resources almost uniformly reference exam findings in white patients.

Case reports on patients with light-complected skin predominate the medical literature on PCD, implicitly making paler skin the default. Cases in patients with darker skin may be missed or misdiagnosed simply because of unfamiliarity with skin that appears more ashen, purple, or hyperpigmented rather than blue.^6^ This patient did not present with the ostensibly pathognomonic “bright blue leg” frequently encountered in both educational resources and clinical imaging series published in academic journals.^7^^,^^8^ Many other clinical exam findings including cyanosis, livedo reticularis, jaundice, and purpura are distinct between patient populations of different skin complexion but are based on a reference frame of patients with lightly complected skin. Expecting to see blue, rather than hyperpigmented, purpuric, or ashen skin can undergird ascertainment bias and diagnostic errors, with delayed disposition and treatment in this and other critical illnesses. In a case report about a patient with toxic epidermal necrolysis, Lester, Taylor, and Chren (2021) describe a patient who spent many hours in the hospital waiting room decompensating, their care delayed because “the characteristic redness” facilitating diagnosis “can be subtle in skin of color.^9^

These subtleties are under-represented in the medical literature. Our literature review yielded 60 case reports on PCD. Only two included images involving patients of color. In a study assessing representation of skin color in dermatology-related Google searches, Kurrti, Austin and Jagdeo et al found that 91.7% displayed light skin.^10^ Although Black people comprise 13.5% of the United States population, Black physicians account for only 5% of the physician workforce in the US as of 2019. People of color more generally account for fewer than one-third of physicians.^11^^,^^12^ Physician demographic disparities derive from multiple interconnected causes including structural and institutional racism, historical segregation in medical education, and marginalization of historically Black medical schools.^7^

The 1910 Flexner Report, which instigated major reforms in medical education in the US, also prompted the closure of all but two historically Black medical schools in an era during which modern biomedical diagnostics were established.^13^ Flexner’s view of the role for Black physicians was providing “hygiene” for Black communities to prevent hookworm and tuberculosis from affecting White populations.^13^ It is no historical accident that many contemporary descriptors for clinical exam findings reflect their presentation in White patients; the privilege of elaborating anatomopathological nomenclature to describe features of disease was deliberately restricted to White physicians whose practice was carried out in racially segregated environments. Consequentially, medical practice today involves unexamined use of terminology that may unintentionally bias the user away from correct diagnosis.

## CONCLUSION

Accurate diagnosis requires recognition of pathology and various diagnostic criteria, which may visually vary significantly between phenotypically distinct populations. Many pathognomonic physical exam findings involve descriptors based on their presentation in White patients. While race is not a biological phenomenon, phenotypic differences confer significant distinctions in manifestation of pathologic findings. Historical racism situating whiteness as default influenced the development of clinical nomenclature, potentially limiting the appropriateness and full diagnostic scope of terms for many clinical exam findings. As a time-sensitive condition, the naming of phlegmasia cerulea dolens can be a disservice to clinicians and patients alike. Emergency clinicians should be conscious of the core features seen in this clinical entity; significant discoloration to an entire extremity with associated pain, swelling, and paresthesia, particularly in a patient with an underlying acquired or inherited thrombophilia.
